# Protocol for the use of Oredsson universal replacement medium for cell banking and routine culturing of monolayer and suspension cultures

**DOI:** 10.1016/j.xpro.2025.103781

**Published:** 2025-04-29

**Authors:** Stina Oredsson, Atena Malakpour-Permlid, Johan Zhu, Tilo Weber

**Affiliations:** 1 Department of Biology, Lund University, 22362 Lund, Sweden; 2 Center for Intelligent Drug Delivery and Sensing Using Microcontainers and Nanomechanics, Department of Health Technology, Technical University of Denmark, 2800 Kongens Lyngby, Denmark; 3 Clinical Microbiology and Infection Prevention and Control, Region Skåne, 22185 Lund, Sweden; 4 Animal Welfare Academy of the German Animal Welfare Federation, 85579 Neubiberg, Germany

**Keywords:** Cell culture, Cell-based assays, Cancer

## Abstract

The Oredsson universal replacement (OUR) medium is the formulation of a universal xeno-free medium designed for the cultivation of human normal and cancer cells in 2D and 3D cultures. Here, we present a protocol for the use of OUR medium for routine culturing, cell banking, and medium modification for suspension culture. We describe steps for thawing, sub-culturing, and freezing cells. We then detail procedures for using these techniques for VERO, A549, THP-1, and Jurkat cells.

## Highlights

• Steps for routine culturing of cells with Oredsson universal replacement (OUR) medium
• Guidelines for thawing cells to obtain high viability and recovery in OUR medium
• Guidelines for freezing cells to maintain viability
• Proof-of-principle studies demonstrated for VERO, A549, THP-1, and Jurkat cells

## Abstract (editor highlights)

Publisher’s note: Undertaking any experimental protocol requires adherence to local institutional guidelines for laboratory safety and ethics.

## Graphical abstract

**Figure:**
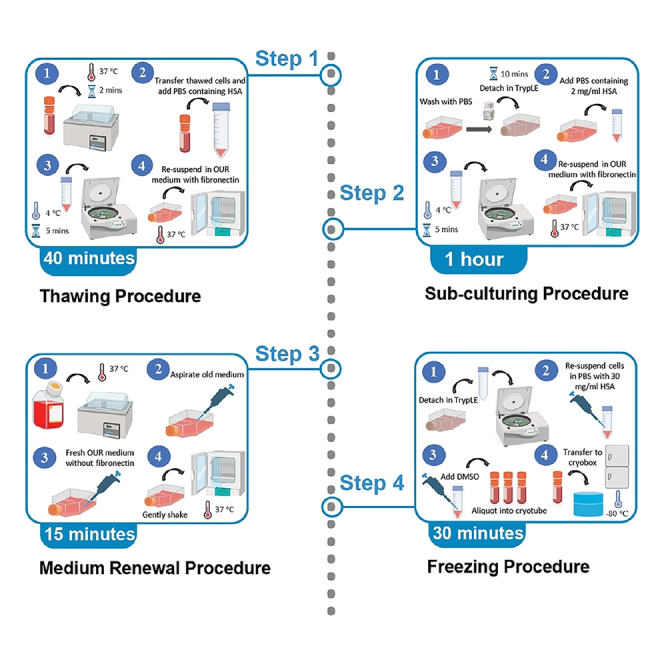

## Before you begin

We have published recipes formulating a universal xeno-free medium designed for the cultivation of human normal and cancer cells in 2D and 3D,^1^^,^^2^ which we call Oredsson universal replacement medium (OUR medium).^3^ Here, we present further protocols for the optimal use of OUR medium. Thus, for a comprehensive understanding of the protocols, we suggest reading our two open access papers and then trying out OUR medium.

OUR medium has an open-source formulation i.e., any laboratory can make it independently. From the beginning, it requires the purchase of a number of different components, which could be expensive if only a small amount of medium is going to be used (for price indications, please see: Table S1. Comparison of cost for different formulations of 500 mL of OUR medium for adherent cells. Table S2. Comparison of cost for different formulations of 500 mL of OUR medium for cells in suspension culture. Table S3. Cost estimation of all individual components of OUR medium). However, different labs can join forces and make the medium together. Currently, a number of xeno-free proprietary media are available on the market. However, it is our notion that the possibility to perform high-quality experiments to comprehensively understand cell biology in different contexts using cells *in vitro* requires full disclosure of cell culture media composition. The section cell lines cultured in OUR medium displays a table with all cell lines that we have routinely cultured in OUR medium so far.

Here, we present detailed protocols for the successful use of OUR medium regarding the thawing of cells, routine culturing as well as the freezing of cells. We also provide some data demonstrating the efficacy of these protocols. In addition, we present a modified version of OUR medium formulated for culturing cells in suspension culture.

### Institutional permissions

Human leukocyte concentrates extracted from human peripheral blood samples purchased from the Medical Service, Laboratory Medicine at Skåne University Hospital in Lund with the permit 2021:18. Informed consent was obtained from healthy blood donors. All experiments involving human blood components were performed with the ethical permit 2010–05769, granted by the Swedish Ethical Review Authority.

### Preparation of PBS containing HSA used in protocols for thawing and freezing of cells and routine passaging

**Timing: 2 h**

The procedures presented below are for solutions used in the protocols described in step-by-step method details.

#### Procedure for preparing 200 mL of PBS containing 30 mg/mL HSA

1. Add 150 mL of sterile PBS (20°C–25°C) to a sterile 500 mL bottle and add 6 g of HSA to it.

***Note:*** The PBS (phosphate-buffered salt solution) used is standard without Ca^2+^ and Mg^2+^. Store PBS at 4°C.

2. Add an additional 50 mL of sterile PBS (20°C–25°C), to achieve a desired concentration of 30 mg/mL HSA.
3. Do not shake. Rock the bottle gently until the HSA is fully dissolved. It may take an hour.
4. Sterile filter the solution using a 0.22 μm filter.
5. Label sterile 50 mL tubes with “Human Serum Albumin, 30 mg/mL PBS”.
6. Aliquot in the sterile tubes (10 mL per tube).
7. Store the tubes at −20°C.

#### Procedure for preparing 400 mL of PBS containing 2 mg/mL HSA

8. Add 300 mL of sterile PBS (20°C–25°C) in a sterile 500 mL bottle and add 1.6 g of HSA to it.
9. Add an additional 100 mL of sterile PBS (20°C–25°C), yielding a desired concentration of 2 mg/mL HSA.
10. Do not shake. Rock the bottle gently until the HSA is fully dissolved. It may take an hour.
11. Sterile filter the solution.
12. Label sterile 50 mL tubes with “Human Serum Albumin, 2 mg/mL PBS”.
13. Aliquot in the sterile tubes (20 mL per tube).
14. Store the tubes at −20°C.

***Note:*** These solutions have been subjected to repeated thaw/freeze cycles (4–5 times) and we have not seen any adverse effects.

**CRITICAL:** According to Biowest (https://biowest.net/p6140-human-serum-albumin-lyophilised/), “**This product is potentially infectious. It is the responsibility of the end user to take all necessary precautions to prevent any contamination of the product or the user themselves.”**

When handling HSA, use gloves, face mask, and a lab coat. Weigh out the compound on a high precision scale placed on a draw bench (or in a fume hood) to prevent inhalation of the compound. Add the PBS to HSA in a LAF bench.

***Alternatives:*** We have also tested HSA from SeraCare, Milford, Massachusetts, USA (https://www.seracare.com/) (see key resources table).

The optimal choice is the use of recombinant HSA (see key resources table).

### Preparation of a solution containing 2 ng IL-2/μL for T cell culturing

**Timing: 1 h**

The procedures presented below are for solutions used in the protocols described in step-by-step method details.

15. Prepare 25 mL of a solution containing 1 mg HSA/mL PBS.
  a. Pipette 12.5 mL PBS to a 50 mL test tube.
  b. Add 12.5 mL of the solution containing 2 mg HSA/mL PBS to the test tube.
  c. Sterile filter the solution and label the test tube: 1 mg HSA/mL PBS.
16. Prepare 5 mL 100 mM acetic acid from 100% acetic acid, which is equivalent to 17.41 M.
  a. Add 4971.3 μL Millipore H_2_O to a test tube labeled 100 mM acetic acid.
  b. Add 28.7 μL of 100% acetic acid to the test tube.
17. Centrifuge the vial with IL-2 at 200–300 *g* before opening in the LAF bench.
18. Reconstitute 10 μg of IL-2 in 50 μL of 100 mM acetic acid.
19. Add 0.450 mL of PBS containing 1 mg HSA/mL to the vial.
20. Transfer the solution to a 15 mL test tube.
21. Add 0.5 mL PBS containing 1 mg HSA/mL to the vial.
22. Transfer to the test tube.
23. Repeat step 7 eight more times until the final volume of 5 mL is reached
24. Sterile filter the final solution.
25. Aliquot the solution in sterile labeled Eppendorf tubes.
26. Store the aliquots at −80°C.

***Note:*** Reconstituted IL-2 can be stored at −80°C for one year according to the manufactureŕs instructions. We recommend using HSA instead of bovine serum albumin as suggested in the manufacturer’s instructions.

### Preparation of OUR medium modified for cells in suspension culture

**Timing: 1 h if all components are made according to Weber et al.**^2^

The procedure presented below is for solutions used in the protocols described in step-by-step method details.

27. Follow the instructions in our previously published paper, 2 with the exceptions stated below.
28. Use RPMI 1640 (see key resources table) instead of DMEM/F12 as the basal medium.
29. Omit all attachment proteins found in our OUR medium i.e., collagen, fibronectin, vitronectin, laminin, and fetuin A.

***Note:*** Omitting the attachment proteins makes the medium cheaper.

## Key resources table

**Table:** 

REAGENT or RESOURCE	SOURCE	IDENTIFIER
**Chemicals, peptides, and recombinant proteins**		

TrypLE Select	Gibco	Cat# A1285901
Dulbecco’s phosphate-buffered saline (DPBS)	Sigma-Aldrich	Cat# D8537
Human serum albumin, from human serum	Biowest	Cat# P6140
Human serum albumin, from human serum	SeraCare	Cat# 1850-0028
Human serum albumin, recombinant	Sartorius	Recombumin
Laminin, recombinant	BioLamina	Cat# CTG-521; Cat# MX-521
Glutamine	Sigma-Aldrich	Cat# G7513-100ML
DMEM/F12 without phenol red	Gibco	Cat# 21041025
RPMI 1640 with phenol red	Sigma-Aldrich	Cat# R0883-500ML
RPMI 1640 without phenol red	Thermo Fisher Scientific	Cat# 11835
Ethanol gradient grade	Sigma-Aldrich	Cat# 1.11727
Recombinant human IL-2	PeproTech	Cat# 200-02
Acetic acid, 100%	Sigma-Aldrich	Cat# 5.43808
Carboxyfluorescein succinimidyl BD Horizon CFSE	BD Biosciences	Cat# 565082
Anti-CD3/CD28 Dynabeads	Thermo Fisher Scientific	Cat# 11161D
Dimethyl sulfoxide (DMSO)	PanReac AppliChem	Cat# A3672

**Experimental models: Cell lines**		

VERO	ATCC	Cat# CRL-1586
A549	ECACC	Cat# 86012804
THP-1	ATCC	Cat# TIB-202
Jurkat	ATCC	Cat# TIB-152
Human leukocyte concentrate	Medical Service, Laboratory Medicine at Skåne University Hospital in Lund with the permit 2021:18 for SO	N/A

**Software and algorithms**		

Software program Hstudio	Phase Holographic Imaging PHI AB, Lund, Sweden	https://phiab.com/
GraphPad Prism	GraphPad Software Inc., Boston, USA	https://www.graphpad.com/
Adobe Illustrator 2024	Adobe, San Jose, CA	https://www.adobe.com/
BioRender	BioRender Inc., Toronto, Ontario, Canada	https://www.biorender.com/

**Other**		

Primaria tissue culture flask, 25 cm^2^	Corning	Cat# 353813
Primaria tissue culture Petri dish, 35 mm	Corning	Cat# 353801
0.22 μm filter	Sarstedt	Cat# 83.1826.001
Hemocytometer, counting chamber, Bürker	Avantor	Cat# HECH40444702
Cryotube	Nunc	Cat# TMO375418
M3 phase holographic microscope	Phase Holographic Imaging 2PHI AB, Lund, Sweden	https://phiab.com/

**Critical commercial assays**		

Naive CD4^+^ T cell isolation kit II	Miltenyi Biotec	http://www.miltenyibiotec.com/ds/130-094-131
Dynabeads human T-activator CD3/CD28	Thermo Fisher Scientific	https://assets.thermoftherm.com/TFS-Assets/LSG/manuals/11131D_32D_61D.pdf
CSFE staining	BD Biosciences	http://www.bdbiosciences.com/content/dam/bdb/products/global/reagents/flow-cytometry-reagents/research-reagents/single-color-antibodies-ruo/565xxx/5650xx/565082_base/pdf/565082.pdf

## Step-by-step method details

The purpose of the procedures is to ensure the correct use of OUR medium for routine culturing procedures such as thawing cells, cell detachment, cell seeding, medium renewal, and freezing cells.

### Procedure for thawing cells

**Timing: 40 min**

The purpose of the protocol is to thaw cells kept in a liquid nitrogen cell bank to obtain a cell suspension with high viability without using any animal-derived products.

1. Thaw PBS containing 30 mg/mL HSA and PBS containing 2 mg/mL HSA and warm to 20°C–25°C.
2. Warm PBS to 20°C–25°C.
3. Heat the water bath to 37°C.
4. Warm OUR medium to 37°C.
5. Fetch the cryotube containing frozen cells.
6. Thaw the cryotube in the water bath for 1.5–2 min until a small amount of ice remains while shaking slowly.
7. Transfer the thawed solution to a sterile 50 mL tube.
8. Add 2 mL PBS containing 30 mg HSA/mL over 2 min while shaking slowly manually (1 mL per minute).
9. Add 8 mL of PBS containing 2 mg HSA/mL over 8 min while shaking slowly (1 mL per minute).

***Note:*** The role of DMSO in preventing ice crystal formation and osmotic effects is well known. Less well-known is the fact that when cells are frozen in a water-containing solution with DMSO, water will be replaced by DMSO in proteins, which will change protein conformation. Thus, a slow reduction of the DMSO concentration will result in a slow replacement of DMSO with water in proteins resulting in a higher degree of correct protein conformation. Therefore, we use a slow rate of addition of a DMSO dilution solution for the first 10 min (steps 8 and 9).

10. Add PBS to a total volume of 40 mL.
11. Centrifuge the cell suspension at 200–300 x *g* for 5 min at 4°C.
12. Aspirate the medium carefully using an aspirator ensuring not to disturb the cell pellet.
13. Gently flick the tube to loosen the pellet and resuspend in 5–7 mL OUR medium.

***Note:*** Choose a volume that will give a good cell count based on how many cells were frozen.

14. Count the cells, presumably using a hemocytometer.
15. Take a sample from the cell suspension for cell counting using a sterile Pasteur pipet.
16. Load cell suspension in the hemocytometer.
17. Count the cells in the predefined grid areas.

***Note:*** If precise viability data of the thawed cells is required, we suggest the use of vital staining methods to accurately assess cell viability.

18. Calculate cell concentration.
19. Seed cells requiring attachment in Corning Primaria tissue culture plastic using OUR medium containing fibronectin.

***Note:*** We suggest the use of Corning Primaria tissue culture plastic when using OUR medium for adherent cells. However, it is the choice of the user to try any other cell culture plastic but keep in mind that the plastic may be the problem if the cells do not attach or take a longer time than usual to attach.

20. Seed cells in suspension culture in OUR medium modified for cells in suspension culture in any tissue culture flask.

***Note:*** The procedure works for cells cryopreserved in a medium containing fetal bovine serum (FBS) and 10% DMSO, as well as for cells frozen using a xeno-free medium as described below.

Originally, we used adaptation protocols to transfer cells from the FBS-supplemented medium to OUR medium.^1^ Presently, we culture cells directly in OUR medium after thawing. We have thawed SH-SY5Y, JIMT-1, HeLa, MCF-7, MCF-10A, HEK, A549, A375, and VERO cells cryopreserved in FBS-supplemented medium as well as the suspension cells Jurkat and THP-1.

**CRITICAL:** Make sure to only warm solutions to 37°C and do not keep solutions a 37°C for more than 10–15 min. Also, while we advise not using phenol red, which displays the pH of the medium, it is highly recommended that the medium is pH equilibrated in a CO_2_ incubator before use.^4^

### Procedure for cell detachment

**Timing: 40 min**

21. Thaw PBS containing PBS containing 2 mg/mL HSA and warm to 20°C–25°C.
22. Use TrypLE Select and PBS at 20°C–25°C.
23. Warm OUR medium to 37°C.
24. Aspirate the medium.
25. Carefully rinse the cell layer with PBS. Use a volume of 0.2 mL/cm^2^ (e.g., 5 mL for a 25 cm^2^ flask).
26. Aspirate the PBS.

***Note:*** Many cell lines do not require rinsing with PBS (step 5) before the addition of TrypLE Select. The medium is just extensively removed. Thus, we suggest the user tries this step with and without rinsing with PBS to gain their own experience.

27. Add TrypLE Select detachment reagent. Use a volume of 40 μL/cm^2^ (e.g., 1 mL for a 25 cm^2^ flask).

***Note:*** TrypZean is another animal-free alternative to TrypLE Select detachment reagent.

28. Ensure even distribution across the cell layer.
29. Incubate at 37°C for 10 min.

***Note:*** Shorter or longer incubation times can be used depending on cell type. Frequent checks under the microscope during the detachment process will help monitor the cell detachment.

30. Look at the cells under a microscope to ensure detachment.
31. If necessary, gently tap or “hit” the flask to further facilitate cells detaching from each other and the surface of the tissue culture vessel.
32. Add 5 mL PBS containing 2 mg/mL of HSA to every mL of TrypLE Select (e.g., 5 mL for a 25 cm^2^ flask or 5 mL of this solution per mL of TrypLE Select).
33. Slowly triturate until obtaining a total mixing of the cell suspension.
34. Transfer the cell suspension to a marked sterile test tube (15 or 50 mL depending on the final volume).
35. Centrifuge at 300 *g* for 5 min at 4°C.
36. Aspirate the supernatant carefully ensuring not to disturb the cell pellet.
37. Carefully resuspend the cells in OUR medium supplemented with fibronectin to support cell attachment.
38. Use a volume that will give an appropriate number of cells per mL to allow cell counting.
39. Take a sample, using a sterile Pasteur pipet, from the cell suspension for cell counting (preferably in a hemocytometer).
40. Load the cell suspension in the hemocytometer.
41. Count the cells in the predefined grid areas and calculate cell concentration.

**CRITICAL:** Our experience is that the TrypLE Select enzyme activity is not inactivated by dilution and subsequent incubation at 37°C. Cell attachment will take considerably longer if TrypLE Select is not removed. After removing TrypLE Select by the centrifugation step, and adding the complete medium, cells in general attach within 1–2 h.

### Procedure for cell seeding using OUR medium supplemented with fibronectin

**Timing: 15 min**

42. First determine the desired number of cells to be seeded per cm^2^ in a tissue culture vessel with a known area and the total number of cells needed for that vessel.
43. Use this information and the cell counting data after detachment to calculate the volume of cell suspension needed to obtain the correct number of cells for seeding.

***Note:*** The volume should be lower than the desired final volume in the vessel.

44. For 25 cm^2^ flasks, use 4–4.5 mL of medium including cell suspension.

***Note:*** The depth of medium in a cell culture vessel should never exceed 2–3 mm, since this can result in insufficient diffusion of O_2_ to the cell layer to compensate for O_2_ use.

45. Add the medium to the flask first.

***Note:*** If possible, add the medium to the vessel and incubate for 10 min to allow binding of attachment proteins to the tissue culture plastic and for temperature and pH equilibration.

46. Add the cell suspension to the flask to obtain the appropriate final volume and cell number per cm^2^.
47. Mix carefully (motion like an eight)
48. Transfer the flask to the CO_2_ incubator (5% CO_2_ in air).
49. Ensure the lid is slightly unscrewed to allow a gas exchange if a flask without a filter is used.

### Procedure for renewal of OUR medium without fibronectin

**Timing: 10 min**

50. Warm the fibronectin free OUR medium to 37°C.
51. Aspirate the old medium without disturbing the cell layer.
52. Add the same volume of OUR medium WITHOUT fibronectin.
53. Ensure the medium is pre-warmed to the appropriate temperature (37°C).
54. Carefully add the medium along the side of the flask to minimize disruption to the cell monolayer.
55. Return the flask to the CO_2_ incubator.
56. Make sure the lid is slightly unscrewed to allow proper gas exchange.

***Note:*** If cells are incubated for one week, we recommend renewing the medium after 3 or 4 days to maintain optimal nutrient levels and remove metabolic waste. Fibronectin is expensive and in our experience, cells do not need fibronectin in a renewal medium. Our notion is that the cell surface will be covered by the fibronectin in the medium used at cell seeding and then no more fibronectin is needed.

### Procedure for freezing cells

**Timing: 30 min**

57. Thaw PBS containing 30 mg/mL of HSA and warm to 20°C–25°C.
58. After detaching cells, determine the total cell number before pelleting (i.e., after Cell detachment step 14).

***Note:*** Use this information for resuspending the cells after pelleting (step 6).

59. Centrifuge at 200–300 x *g* for 5 min at 4°C.
60. Aspirate the supernatant carefully, ensuring that you do not disturb the cell pellet.
61. Resuspend the pelleted cells in PBS containing 30 mg/mL HSA at the concentration of 2 × 10^6^ cells per 1425 μL.
62. Mark cryotubes with cell line, passage number, and date.
63. Carefully add 75 μL DMSO per 1425 μL of cell suspension in PBS containing 30 mg/mL HSA.
64. Mix carefully. This results in a DMSO concentration of 5%.
65. Immediately, transfer the cell suspension to the marked cryotube (1.5 mL per tube).
66. Immediately proceed to transfer the cryotube into a cell freezing container for 2 mL cryogenic vials.
67. Put the container in a −80°C freezer.
68. Leave it undisturbed for at least 24 h.
69. Transfer to a liquid nitrogen tank for long-term storage.

### Proof-of-principle procedure: Thawing and freezing of VERO and A549 cells

**Timing: Please see above for freezing and thawing**

70. Thaw VERO cells ordered from ATCC (see key resources table) or A549 cells ordered from ECACC (see key resources table) according to the Protocol for Thawing Cells.
71. Seed all thawed cells into a 25 cm^2^ Primaria cell culture flask labeled with VERO or A549, p1 (passage 1, for the passaging in the lab), and date.

***Note:*** Regarding passage number, even though we recommend p1 after thawing and seeding in OUR medium, it should be remembered that the cells obtained may have had another passage number related to passaging in another medium.

72. Incubate the cells for 4 days.
73. Detach according to the Cell detachment protocol.
74. Count the number of cells.
75. Seed cells at a density of 25000 cells/cm^2^ in the required number of flasks (depending on the surface area used).
76. Labeling the flasks: VERO or A549, p2, and date.
77. Change medium after 3–4 days (fibronectin free).
78. Detach cells 7 days after seeding and repeat the routine.
79. Use cells for experiments (see growth curve with VERO and A549 cells in OUR medium).

**CRITICAL:** At some point, freeze cells according to the Protocol for Freezing Cells to have a frozen stock.

### Proof of principle: Growth curve with VERO and A549 cells in OUR medium

**Timing: 60 min each day**

80. Label 12 Primaria Petri dishes with 3.5 cm diameter (9.6 cm^2^ area) with VERO or A549.
81. Add 2.5 mL of OUR medium containing fibronectin to each Petri dish.
82. Place the dishes in the 5% CO_2_ incubator for equilibration for an hour.

***Note:*** Use a plastic tray for easier handling.

83. Detach VERO or A549 cells according to the Cell detachment protocol.
84. Determine the cell concentration.
85. Prepare 15 mL of cell suspension with 0.15 × 10^6^ cells per 0.5 mL of medium in a sterile 50 mL tube.

***Note:*** The cell seeding information yields 15,600 cells/cm^2^.

86. Take the equilibrated Petri dishes from the incubator and place them in the LAF bench.
87. Mix the cell suspension in the 50 mL tube by carefully inverting it 6 times.
88. Add 0.5 mL of the cell suspension (containing 0.15 × 10^6^ cells) to each Petri dish.
89. Carefully shake the Petri dishes in an 8 motion to obtain even cell distribution.

***Note:*** This step is more efficient if the Petri dishes are placed on a tray.

90. Transfer the Petri dishes to the incubator.
91. Determine the cell number in three Petri dishes every day for 4 days.

***Note:*** Use the same cell counting method (hemocytometer or cell counter) throughout the experiment to ensure consistency.

92. Detach the cells.
93. Count the cells in a hemocytometer.

***Note:*** We prefer the hemocytometer as it involves looking at the cells in the microscope and learning what a cell type looks like after detachment and also how cell sizes change with the growth pattern (cell cycle-related) and treatment.

**Figure 1:**
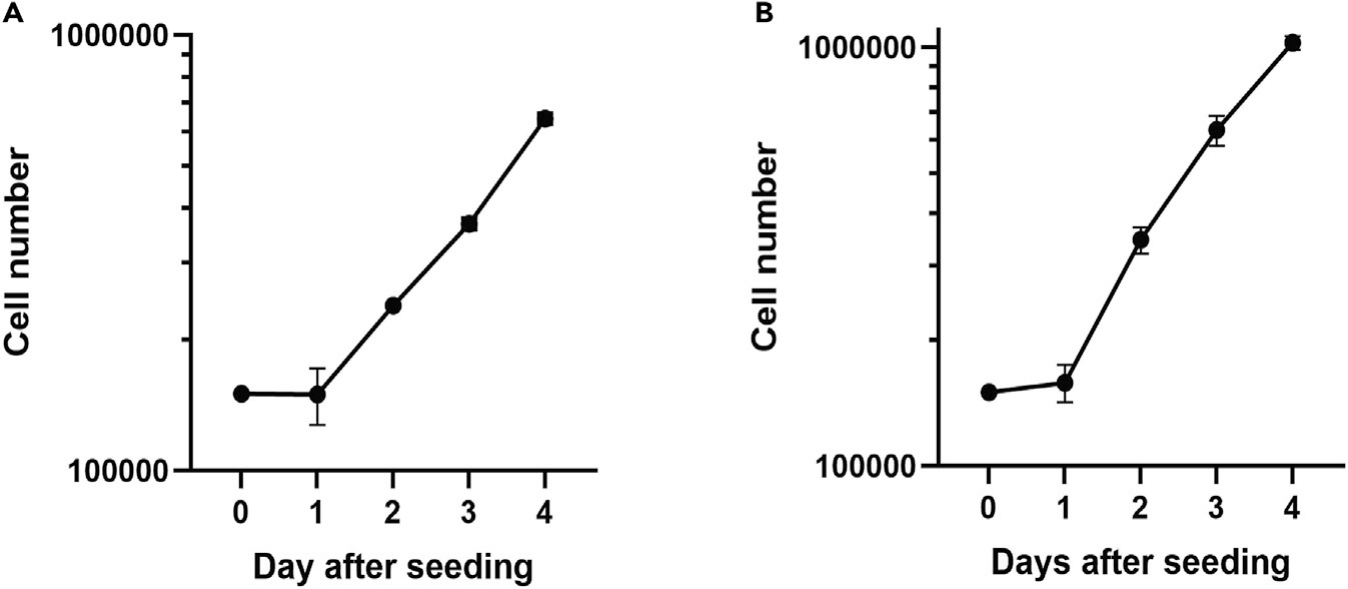
Growth curves for VERO and A549 cells (A) VERO and (B) A549 cells cultured in OUR medium. The symbols represent the mean of the cell number in three Petri dishes and the bars ± SD.

94. Use the collected data to draw a growth curve (see Figure 1 below).

### Proof-of-principle procedure: Growth curves for THP-1 and Jurkat cells in OUR medium modified for suspension culture

**Timing: 60 min each day**

The formulation of OUR medium for suspension culture is found above.

95. Label 3 tissue culture flasks with an area of 25 cm^2^ with the cell line name and the numbers 1, 2, or 3.
96. Add 4 mL of OUR medium for suspension culture to each flask.
97. Put them in the incubator for 30 min to equilibrate the medium with respect to temperature and pH. Remember to loosen the screw cap of the flasks.
98. Determine the cell concentration in routine cultures of THP-1 or Jurkat cells.

***Note:*** A sample can be easily taken from a cell suspension using a sterile Pasteur pipette. By dipping the pipette into the cell suspension, capillary action will draw up a small volume that can be applied directly to a hemocytometer for counting.

**Figure 2:**
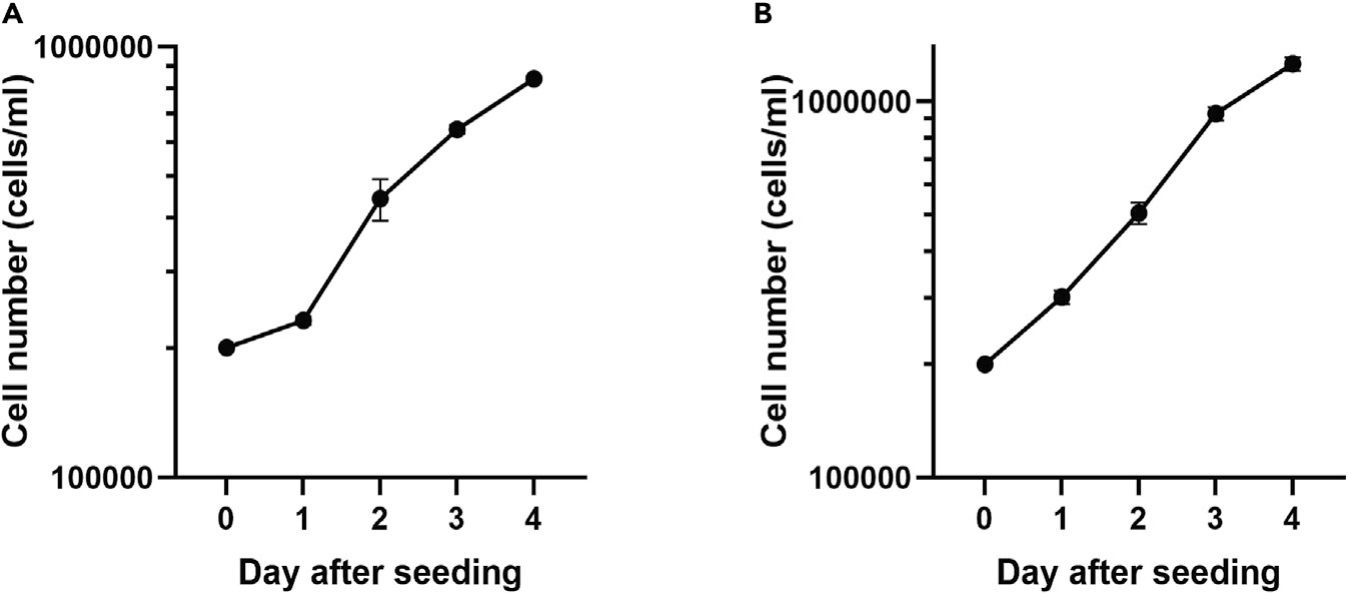
Growth curves for THP-1 and Jurkat cells (A) THP-1 cells and (B) Jurkat cells cultured in OUR medium modified for suspension culture. The symbols represent the mean of the cell number in three flasks and the bars ± SD.

***Note:*** An individual growth curve can be drawn for each flask.

***Note:*** THP-1 and Jurkat cells, frozen in ampoules with 10% FBS and 10% DMSO, were thawed according to the Protocol for Thawing Cells described earlier. After thawing, the cells were seeded at a density of 0.2–0.3 × 10^6^ cells/mL in our medium for suspension culture. A cell bank was established following the Protocol for freezing cells as described above.

99. Prepare 5 mL of a cell suspension with 1 × 10^6^ cells/mL OUR medium for suspension culture.
100. Take the flasks prepared with medium from the incubator.
101. Add 1 mL cell suspension to each flask.
102. Mix carefully and put in the incubator. Remember to loosen the screw cap of the flasks.
103. On days 1-4 after seeding, take out one flask at a time and take a sample from the cell suspension (after careful mixing) using a sterile Pasteur pipet for cell counting in a hemocytometer.
104. Return the flasks with the remaining cell suspension to the incubator immediately.
105. Use the collected data to draw a growth curve (see Figure 2 below).

### Proof-of-principle procedure: CD4^+^ T cell proliferation in OUR medium modified for suspension culture

#### Day 1

**Timing: 3 h**

106. Isolate Naïve CD4^+^ T cells from human leukocyte concentrate according to a standard isolation procedure (see key resources table).
107. Warm 10 mL of OUR medium modified for suspension culture to 37°C.
108. Seed the isolated CD4^+^ T cells at a density of 0.5 × 10^6^ cells/mL of OUR medium modified for cells in suspension culture.
109. Supplement the medium with 1 ng/mL of IL-2.
110. Incubate the cells in a 5% CO_2_ incubator for 24 h.

#### Day 2

**Timing: 3 h**

111. Prepare anti-CD3/CD28 Dynabeads according to the manufacturer’s protocol (see key resources table).
112. Stain the CD4^+^ T cells with CFSE according to the manufacturer’s protocol (see key resources table).
113. Resuspend them in OUR medium modified for cells in suspension culture (37°C) at the desired concentration.
114. Transfer the CFSE-stained CD4^+^ T cells to the desired tissue culture plate.
115. Add the prepared anti-CD3/CD28 Dynabeads at the concentration suggested by the manufacturer.
116. Remember to have a negative control, i.e., without Dynabeads.
117. Incubate at 37°C protected from light covered with aluminum foil.

#### Day 6 or 7

**Timing: 3 h**

118. After 4–5 days of incubation, remove the T cell suspension.
119. Run the sample in a flow cytometer for analysis of CFSE fluorescence.

***Note:*** We have used a BD Accuri C6 flow cytometer (BD Biosciences, San Jose, CA, USA).

***Note:*** There are several proprietary xeno-free media on the market that support T cell proliferation.^5^^,^^6^ However, OUR medium modified for suspension culture is open access. Thus, the composition is known, which always is an advantage.

## Expected outcomes

**Table:** Cell lines cultured in OUR medium

Cell line name	Characterization	Culture type	Provider	Identifier
CaCo-2	Human colon cancer cells	Adherent	ATCC a	HTB-37
Cancer-associated fibroblasts	Human fibroblasts	Adherent	Kojima et al., 2010^7^	—
JIMT-1	Human breast cancer cells	Adherent	DSMZ^b^	ACC589
KeratinoSens	Human keratinocytes	Adherent	acCCELLerate^c^	RE242
L929	Mouse fibroblasts	Adherent	ATCC	CCL-1
MDA-MB-231	Human breast cancer cells	Adherent	ATCC	HTB-26
MiaPaCa-2	Human pancreatic cancer cells	Adherent	ATCC	CRL-1420
AsPC-1	Human pancreatic cancer cells	Adherent	ATCC	CRL-1682
BxPC-3	Human pancreatic cancer cells	Adherent	ATCC	CRL-1687
C6	Rat glioma cells	Adherent	ATCC	CCL-107
CaOv-3	Human ovarian cancer cells	Adherent	ATCC	HTB-75
SW626	Human ovarian cancer cells	Adherent	ATCC	HTB-78
HDF, adult	Human dermal fibroblasts, adult	Adherent	Sigma-Aldrich	106-05A
HeLa	Human cervical cancer cells	Adherent	ATCC	CRM-CCL-2
LAN-1	Human neuroblastoma cells	Adherent	DSMZ	ACC 655
SH-SY5Y	Human neuroblastoma cells	Adherent	ATCC	CRL-2266
MCF-7	Human breast cancer cells	Adherent	ATCC	HTB-22
MCF-10A	“Normal-like” human breast epithelial cells	Adherent	ATCC	CRL-10317
NmuMg	Mouse mammary gland epithelial cells	Adherent	ATCC	CRL-1636
PanC-1	Human pancreatic cancer cells	Adherent	ATCC	CRL-1469
A549	Human lung epithelial cells	Adherent	ECACC^d^	86012804
VERO E6	Green monkey kidney cells	Adherent	ATCC	CRL-1586
HEK293	Human embryo kidney cells	Adherent	ATCC	CRL-1573
A375	Human melanoma cells	Adherent	ATCC	CRL-1619
THP-1	Human monocytic cells	Suspension	ATCC	TIB-202
Jurkat	Human T lymphocyte cells	Suspension	ATCC	TIB-152
Human T cells^e^		Suspension		

a American Type Culture Collection, Manassas, Virginia, United States of America. https://www.atcc.org/.

b Deutsche Sammlung von Mikroorganismen und Zellkulturen (German Collection of Microorganisms and Cell Cultures), Braunschweig, Germany. https://www.dsmz.de/.

c Hamburg, Germany. https://www.accellerate.me/.

d European Collection of Authenticated Cell Cultures, Porton Down, United Kingdom of Great Britain and Northern Ireland. https://www.culturecollections.org.uk/.

e Isolated from human leukocyte concentrate from Medical Service, Laboratory Medicine at Skåne University Hospital in Lund, Sweden. See Institutional permission above.

### Growth curve of VERO cells and A549 in OUR medium

After a 24 h lag period, the VERO cells grow exponentially with a population doubling time (PDT) of about 34 h (Figure 1A). A population doubling time of 24 h has been reported for VERO cells in a medium supplemented with 10% FBS.^8^

In general, cells are only observed statically in a phase contrast microscope, and therefore limited information about cell behavior in between microscopic evaluations is less known. To address this, we have studied cell behavior for several days by time-lapse imaging using an M3 holographic microscope (PHI AB, Lund, Sweden) equipped with a 10X phase contrast objective. Methods Video S1 shows a phase contrast microscopy time-lapse video of VERO cells cultured in OUR medium with HSA from human serum (related to 70–94). Images were captured every 10 min for 68 h.

Methods Video S1. Phase contrast microscopy time-lapse video of VERO cells cultured in OUR medium with HSA from human serum

Methods Video S2 shows a phase contrast microscopy time-lapse video of VERO cells cultured in OUR medium with human recombinant HSA (Recombumin from Sartorius) instead of HSA from human blood (related to 70–94). Images were captured every 10 min for 72 h.

Methods Video S2. Phase contrast microscopy time-lapse video of VERO cells cultured in OUR medium with human recombinant HSA (Recombumin from Sartorius)

Figure 1B shows a growth curve for A549 human lung cancer cells with a 24 h lag phase before commencement of exponential cell proliferation. The PDT during exponential growth is around 27 h. The population doubling time for A549 cells in medium supplemented with 10% FBS has been reported to be around 21–22 h^9^ Cellosaurus reports population doubling times between 18 and 37 h (www.cellosaurus.org). When grown in the proprietary media X-VIVO or CnT-PR-A, population doubling times of 33 and 51 h have been reported, respectively.

Methods Video S3 shows a phase contrast microscopy time-lapse video of A540 cells cultured in OUR medium with HSA from human serum (related to 70–94). Images were captured every 10 min for 94 h.

Methods Video S3. Phase contrast microscopy time-lapse video of A540 cells cultured in OUR medium with HSA from human serum

Methods Video S4 shows a phase contrast microscopy time-lapse video of A540 cells cultured in OUR medium with human recombinant HSA (Recombumin from Sartorius) instead of HSA from human blood (related to 70–94). Images were captured every 10 min for 88 h.

Methods Video S4. Phase contrast microscopy time-lapse video of A540 cells cultured in OUR medium with human recombinant HSA (Recombumin from Sartorius)

### Growth curves of THP-1 and Jurkat cells in OUR medium modified for suspension culture

After a 24 h lag period, the THP-1 cells start proliferating very rapidly and the shortest population doubling time is between day 1 and day 2 after seeding (25.6 h) after which the PDT declined (44.8 h between days 2 and 3 and 61.5 h between days 3 and 4). The maximum density of THP-1 cells we have obtained is around 1.2 × 10^6^ cells/mL, which is similar to that reported by ATCC (www.atcc.org/products/tib-202). Cellosaurus reports PDTs between 26 and 50 h for THP-1 cells cultured in a medium supplemented with 10% FBS (www.cellosaurus.org).

Methods Video S5 shows a phase contrast microscopy time-lapse video of THP-1 cells cultured in OUR medium for suspension cells with HSA from human serum (related to 95–105). Images were captured every 10 min for 77 h.

Methods Video S5. Phase contrast microscopy time-lapse video of Jurkat cells cultured in OUR medium with HSA from human serum

The lag phase is almost absent for Jurkat cells (Figure 2B) and the PDT is about 30 h between day 1 and day 3 after seeding. Cellosaurus reports a population doubling times of 26–35 h for Jurkat cells cultured in a medium with 10% FBS (www.cellosaurus.org).

Methods Video S6 shows a phase contrast microscopy time-lapse video of Jurkat cells cultured in OUR medium for suspension cells with HSA from human serum (related to 95–105). Images were captured every 10 min for 92 h.

Methods Video S6. Phase contrast microscopy time-lapse video of THP-1 cells cultured in OUR medium with HSA from human serum

### Proliferation of CD4+ T cells in OUR medium modified for suspension culture

**Figure 3:**
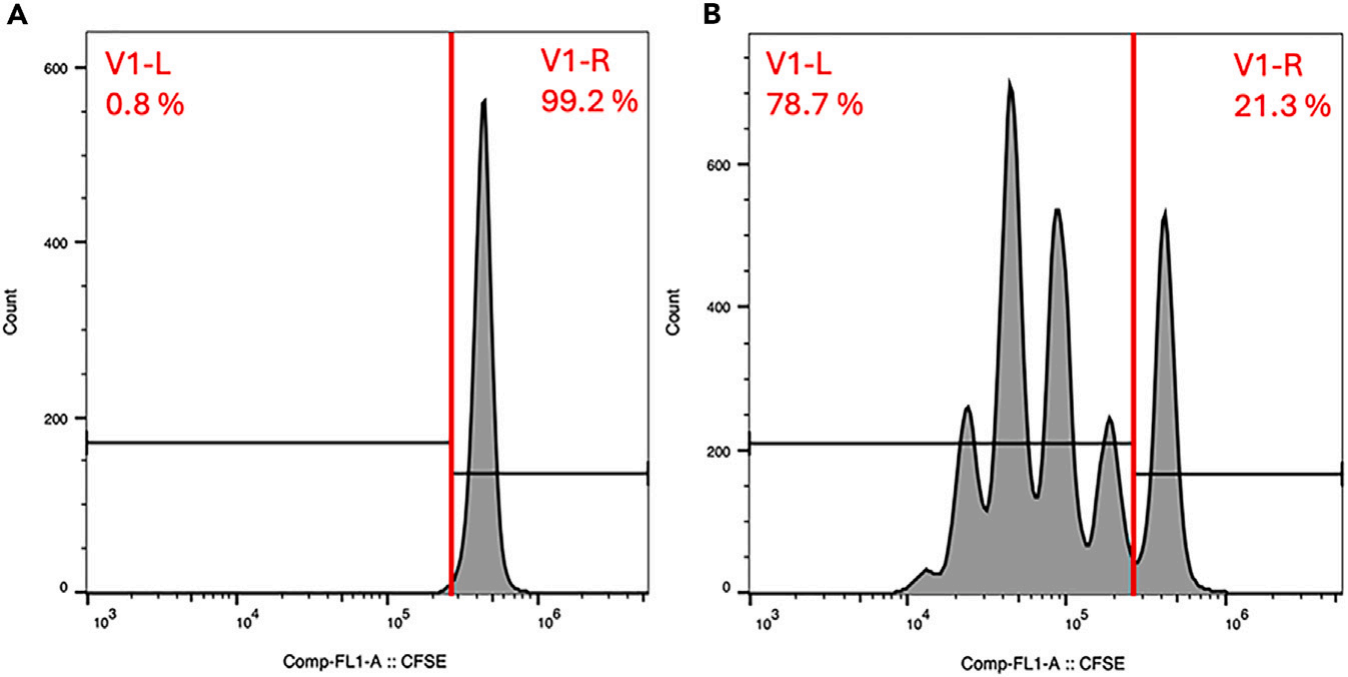
Representative flow-cytometry-derived data of T cells seeded in OUR medium modified for suspension culture after staining with CFSE and incubated for four days (A) T cells cultured in the absence of anti-CD3/CD28 Dynabeads show a single peak, indicating no cell division. (B) T cells stimulated with anti-CD3/CD28 Dynabeads display four major peaks to the left of the red line, reflecting four population doublings. The data are representative of six independent samples from two independent experiments.

Figure 3 shows CFSE-labeled CD4^+^ T cells seeded in OUR medium modified for suspension culture, either in the absence (Figure 3A) or presence (Figure 3B) of anti-CD3/CD28 Dynabeads. The data show that T cell proliferation is supported in OUR medium (related to 106–119).

We also used an ELISA-based method to measure interferon-g (IFN-g) in the cell culture medium of the cells sampled for flow cytometry. In the absence of anti CD3/CD28 Dynabead stimulation, there was no detectable IFN-g in the medium. However, when the CD4^+^ T cells were induced to proliferate, the IFN-g concentration was 61.9 ± 9.9 pg/mL (mean ± SD of *n* = 6 independent samples from two independent experiments). Thus, OUR medium modified for suspension culture supports both the proliferation of T cells and the production of IFN-g.

## Limitations

A limitation presently is that OUR medium is not yet commercially available. However, since it has an open access formulation, anybody can prepare it. The medium is easy to craft by following the point-by-point instructions in our previous work.^2^ Table S1–S3 provides a compilation of the costs for all components of OUR medium, including product identifiers and suppliers. Some readers may find it a limitation that we have not provided a direct comparison to media supplemented with FBS in our studies. However, our main aim has been to adapt cell lines to xeno-free protocols for thawing, routine culturing, and freezing. Regarding the majority of cell lines cultured in OUR medium, we have concentrated on routine culturing and establishing consistent routine growth, which is the basis of good and robust experimental work. We have chosen not to compare our results with the FBS-supplemented medium, as FBS should not be considered the gold standard in research. On the other hand, there is extensive data already available in the literature for cells grown in FBS-supplemented medium, which provides a well-established reference for comparisons.

## Troubleshooting

### Problem 1

The complete cell culture media are stable for 2 months when stored at 4°C. It may be stable for a longer time, but we have never investigated this.

### Potential solution

• If the rate of cell proliferation reduces, check the medium age.
• Always indicate the date and label the medium flask after its preparation.

### Problem 2

Note that OUR medium does not contain the xenobiotic HEPES for pH stabilization. Thus, as the medium is stored in a bottle with an atmosphere of 0.04% CO_2_, the pH of the medium will gradually increase. Some cell types are very sensitive to basic media, i.e., a pH higher than 7.4. A high protein content media such as when supplementing medium with 10% FBS (yielding 4–5 mg bovine serum albumin/mL medium) can partially protect cells. OUR medium has a lower protein content (1.25 mg/mL) compared to medium supplemented with 10% FBS, and therefore cells become more sensitive to basic pH.

### Potential solution

• To prevent this, it is highly recommended to equilibrate the medium in the CO_2_ incubator for 30 min before use.
• Store the medium properly by keeping bottles tightly sealed minimizing exposure to air to reduce CO_2_ loss.
• Monitor pH regularly using a sterile pH meter to detect any pH fluctuations before cell culture use.

### Problem 3

The cells take very long to attach.

### Potential solution

• Check the cell culture plastic.
• Cells may attach to negatively charged polystyrene, but our experience is that cell attachment occurs much faster using Corning Primaria tissue culture plastic.

## Resource availability

### Lead contact

Questions regarding further information should be directed to the lead contact, Stina Oredsson (stina.oredsson@biol.lu.se).

### Technical contact

Questions about the technical specifics of performing the protocols should be directed to the technical contact, Stina Oredsson (stina.oredsson@biol.lu.se).

### Materials availability

The media used in this publication are available upon request.

### Data and code availability

All primary data are available upon request.

## Acknowledgments

This research could not have been done without funding from Forska Utan Djurförsök, Stockholm, Sweden (https://forskautandjurforsok.se/, grant number F2020-002) and donations through crowdfunding by Carolina le Prince and the Kalenderflickorna (https://www.lu.se/artikel/kalenderflickor-i-cancerforskningens-tjanst/); Bröstcancerföreningen Pärlan Helsingborg, Ramlösa, Sweden (https://helsingborg.brostcancerforbundet.se/); Bröstcancerföreningen Viktoria Ängelholm, Hjärnarp, Sweden (https://angelholm.brostcancerforbundet.se/); and Stig and Lisa Ekelund and Mari-Ann and Brainerd Lindberg via Lund University Development Office, Lund, Sweden. The sponsors have no other role than providing funding. We acknowledge the use of the BioRender platform (http://www.BioRender.com) for creating the graphical abstract in this publication. We thank Agnieszka Czopek for expert technical help regarding the growth curves.

## Author contributions

S.O.: conceptualization, methodology, funding acquisition and resources, and writing – review and editing. A.M.-P.: conceptualization, visualization, and writing – review and editing. J.Z.: methodology (T cells) and writing – review and editing. T.W.: conceptualization and writing – review and editing.

## Declaration of interests

The authors declare no competing interests.
