# Supplementary material for: Protocol for the use of Oredsson universal replacement medium for cell banking and routine culturing of monolayer and suspension cultures
Source: STAR Protoc. 2025 Apr 29;6(2):103781. doi: 10.1016/j.xpro.2025.103781 (PMC12076805; doi:10.1016/j.xpro.2025.103781)
Supplement: Supplementary file 7 — Document S1. Tables S1–S3 [file mmc1.pdf]

# Supplemental information

## Supplemental tables

Table S1 shows a summary of the costs for OUR medium with or without fibronectin, as well as when supplementing with recombinant HSA and laminin. When seeding cells after detachment, a medium supplemented with fibronectin is used (column "A with fibronectin" in Table S1). However, when changing the medium between passaging, a medium without fibronectin is used (column "A without fibronectin" in Table S1). Medium A is composed of HSA isolated from human blood and laminin from human placenta, however, they can be replaced with recombinant proteins resulting in a slightly more expensive medium (column "B with fibronectin" and "B without fibronectin"). The cost of 500 ml medium supplemented with 10 % high-quality FBS would be between 90-110 Euro, depending on other additions to the medium.

Table S1. Comparison of cost for different formulations of 500 ml of OUR medium for adherent cells. [a]

| | A with fibronectin [b] | A without fibronectin [c] | B with fibronectin [d] | B without fibronectin [c] |
|---|---|---|---|---|
| OUR medium | 141 Euro | 97 Euro | 163 Euro | 119 Euro |

[a] Costs in EURO, based on Swedish list prices October 1, 2024. 1 SEK = 0.088 EUR.

[b] In the original OUR medium, human serum albumin (HSA) obtained from human blood and laminin obtained from human placenta are used. [1,2]

[c] When an old medium is replaced, fibronectin free medium should be used.

[d] Recombinant HSA instead of HSA derived from human blood. Recombinant laminin instead of laminin from the human placenta. (please see the Key resources table).

Table S2 shows a summary of the costs for OUR medium modified for suspension culture with HSA isolated from human blood (A) or recombinant HSA (B). This modified medium is based on RPMI1640 instead of DMEM/F12 and includes the same components as the original OUR medium, but lacks the attachment proteins (fibronectin, collagen, laminin, fetuin A, and vitronectin).

Table S2. Comparison of cost for different formulations of 500 ml of OUR medium for cells in suspension culture (OUR medium S). [a]

| | A [b] | B [c] |
|---|---|---|
| OUR medium S | 73 Euro | 100 Euro |

[a] Costs in EURO, based on Swedish list prices October 1, 2024. 1 SEK = 0.088 EUR.

[b] In the original OUR medium S we use human serum albumin (HSA) obtained from human blood. The medium does not contain attachment proteins.
[c] Recombinant HSA instead of HSA from human blood.

Table S3. Cost estimation of all individual components of OUR medium.

| Product | Provider | Product identification | EUR [1] | Volume/amount in 500 ml | Cost for 500 ml (EUR) |
|---|---|---|---|---|---|
| DMEM/F12 500 ml | Sigma-Aldrich | L0090-500ML | 23.94 | 500 ml | 23.94 |
| L-Glutamine | Sigma-Aldrich | G7513-100ML | 31.42 | 5 ml | 1.5708 |
| Non-essential amino acids | Sigma-Aldrich | M7145-100ML | 32.47 | 5 ml | 1.6236 |
| Sodium pyruvate | Sigma-Aldrich | S8636-100ML | 18.22 | 5 ml | 0.9108 |
| Penicillin-Streptomycin | Sigma-Aldrich | P0781-100ML | 15.84 | 5 ml | 0.792 |
| All-*trans*-retinoic acid | Sigma-Aldrich | R2625-50MG | 55.79 | 12.5 µg | 0.013948 |
| β-Estradiol | Sigma-Aldrich | E2758-250MG | 33.09 | 250 ng | 0.000033088 |
| Hydrocortisone | Sigma-Aldrich | H0888-1G | 52.18 | 125 ng | 0.000006523 |
| Triiodothyronine | Sigma-Aldrich | T6397-100MG | 88.88 | 0.1 µg | 0.00008888 |
| **Ethanol solution** [2] | | | | | |
| 4-Aminobenzoic acid | Sigma-Aldrich | A9878-5G | 18.66 | 6 µg | 0.00002239 |
| Cholesterol | Sigma-Aldrich | C3045-5G | 102.10 | 25 µg | 0.0005104 |
| Lipoic acid | Sigma-Aldrich | 07039-10MG | 43.38 | 25 µg | 0.10846 |
| Linoleic acid | Sigma-Aldrich | L1012-100MG | 32.82 | 500 µg | 0.16412 |
| **NaOH solution** [3] | | | | | |
| Folic acid | Sigma-Aldrich | F8758-5G | 45.58 | 165 µg | 0.001504272 |
| Uracil | Sigma-Aldrich | U1128-25G | 52.18 | 37.5 µg | 0.000078276 |
| Xanthine | Sigma-Aldrich | X3627-1G | 88.88 | 42.5 µg | 0.0037774 |
| **$H_2O$ solution** [4] | | | | | |
| Ascorbic acid | Sigma-Aldrich | A4403-100MG | 124.96 | 6 µg | 0.0074976 |
| Choline chloride | Sigma-Aldrich | C7527-100MG | 51.83 | 1.75 mg | 0.00090706 |
| Glutathione | Sigma-Aldrich | G6013-5G | 136.40 | 6 µg | 0.00016368 |
| *I*-inositol | Sigma-Aldrich | I7508-50G | 74.89 | 2.25 mg | 0.00336996 |
| *O*-Phosphorylethanolamine | Sigma-Aldrich | P0503-5G | 43.03 | 2.5 mg | 0.021516 |
| Ribose | Sigma-Aldrich | R9629-25G | 154.88 | 62.5 µg | 0.0003872 |
| Selenous acid | Sigma-Aldrich | 211176-10G | 32.91 | 4 µg | 0,0000132 |
| Thiamine hydrochloride | Sigma-Aldrich | T1270-25G | 56.32 | 40 µg | 0.000090112 |

| | | | | | |
|---|---|---|---|---|---|
| α-Tocopherol phosphate | Sigma-Aldrich | T2020-250MG | 161.04 | 1.5 µg | 0.00096624 |
| Vitamin B12 | Sigma-Aldrich | V6629-100MG | 73.22 | 175 µg | 0.128128 |
| **Carrier Proteins** | | | | | |
| Transferrin (recombinant) | Sigma-Aldrich | T3705-1G | 564.08 | 25 mg | 0.014102 |
| Human serum albumin (human blood) [5] | Seracare | 1850-0028-100G | 699.60 | 625 mg | 4.37 |
| **Attachment proteins** | | | | | |
| Fibronectin (human plasma) | EMD Millipore Corporation | FC010-10MG | 670.56 | 655 µg | 43.92168 |
| Collagen 4 (human plasma) | Sigma-Aldrich | C5533-5MG | 179.52 | 50 µg | 1.79 |
| Laminin (human placenta) [6] | Sigma-Aldrich | L6274-.5MG | 563.2 | 10 µg | 11.26 |
| Fetuin A (human plasma) | Sigma-Aldrich | G0516-1MG | 404.80 | 20 µg | 8.10 |
| Vitronectin (recombinant) | Stem Cell Technologies | 7180-2ML (250UG/ML) | 76.74 | 50 µg | 7.67 |
| **Growth factor proteins** | | | | | |
| Insulin (recombinant) | Sigma-Aldrich | I9278-5ML | 200.64 | 100 µl | 4.01 |
| Insulin-like growth factor 1 (recombinant) | Thermofisher Scientific | PHH0071-100UG | 296.56 | 2.5 µg | 7.41 |
| Basic fibroblast growth factor (recombinant) | Sigma-Aldrich | F3685-25UG | 372.24 | 0.5 µg | 7.44 |
| Epidermal growth factor (recombinant) | Sigma-Aldrich | E9644-.2MG | 283.36 | 10 µg | 7.08 |
| Platelet-derived growth factor AA (recombinant) | PeproTech | 100-13A-50UG | 418.00 | 10 µg | 8.36 |

[1] Costs in EURO, based on Swedish list prices October 1, 2024. 1 SEK = 0.088 EUR.

[2] Found in Ethanol solution (Weber et al., 2024).

[3] Found in NaOH solution (Weber et al., 2024).

[4] Found in $H_2O$ solution (Weber et al., 2024).

[5] Alternative: Human serum albumin (recombinant), Sartorius, Recombumin (10 %, 50 ml). Cost for 625 mg for 500 ml: 30.80 EUR.

[6] Alternative: 1. Biolamina, CT-521 (clinical grade)-500 µg. Cost for 10 µg for 500 ml: 8.59 EUR. 2. Biolamina, MX-512 (pre-clinical grade)-500 µg. Cost for 10 µg for 500 ml: 7.98 EUR.
